# Fas/FasL Pathway Participates in Regulation of Antiviral and Inflammatory Response during Mousepox Infection of Lungs

**DOI:** 10.1155/2015/281613

**Published:** 2015-03-22

**Authors:** Karolina Bień, Justyna Sokołowska, Piotr Bąska, Zuzanna Nowak, Wanda Stankiewicz, Malgorzata Krzyzowska

**Affiliations:** ^1^Military Institute of Hygiene and Epidemiology, Kozielska 4, 01-163 Warsaw, Poland; ^2^Department of Morphological Sciences, Faculty of Veterinary Medicine, Warsaw University of Life Sciences, Ciszewskiego 8, 02-786 Warsaw, Poland; ^3^Department of Preclinical Sciences, Faculty of Veterinary Medicine, Warsaw University of Life Sciences, Nowoursynowska 159, 02-776 Warsaw, Poland; ^4^Department of Genetics and Animal Breeding, Faculty of Animal Science, Warsaw University of Life Sciences, Ciszewskiego 8, 02-786 Warsaw, Poland

## Abstract

Fas receptor-Fas ligand (FasL) signalling is involved in apoptosis of immune cells as well as of the virus infected target cells but increasing evidence accumulates on Fas as a mediator of apoptosis-independent processes such as induction of activating and proinflammatory signals. In this study, we examined the role of Fas/FasL pathway in inflammatory and antiviral response in lungs using a mousepox model applied to C57BL6/J, B6. MRL-Faslpr/J, and B6Smn.C3-Faslgld/J mice. Ectromelia virus (ECTV) infection of Fas- and FasL-deficient mice led to increased virus titers in lungs and decreased migration of IFN-*γ* expressing NK cells, CD4+ T cells, CD8+ T cells, and decreased IL-15 expression. The lungs of ECTV-infected Fas- and FasL-deficient mice showed significant inflammation during later phases of infection accompanied by decreased expression of anti-inflammatory IL-10 and TGF-*β*1 cytokines and disturbances in CXCL1 and CXCL9 expression. Experiments in vitro demonstrated that ECTV-infected cultures of epithelial cells, but not macrophages, upregulate Fas and FasL and are susceptible to Fas-induced apoptosis. Our study demonstrates that Fas/FasL pathway during ECTV infection of the lungs plays an important role in controlling local inflammatory response and mounting of antiviral response.

## 1. Introduction

Fas receptor (CD95; tumor necrosis factor receptor superfamily member 6, TNFRSF6) is a member of the family of tumor necrosis factor receptors (TNF) and, more specifically, the death receptor subfamily. It contains a death domain (DD death domain), which is required to initiate apoptosis [1]. Interactions between Fas and its ligand, FasL, lead to induction of apoptosis in sensitive cells. Fas-induced apoptosis is involved in the cytotoxic activity of T cells [2] and NK cells [3] and plays a key role in maintaining the immune tolerance [4, 5]. Previous studies have demonstrated that mice with inactivating mutations in FAS (lpr) or FASL (gld) have trouble controlling infections with West Nile virus [6], influenza virus [7], herpes simplex virus-1 [8], herpes simplex virus-2 [9], and mouse hepatitis virus [10].

Ectromelia virus (ECTV; family Poxviridae, genus* Orthopoxvirus*) is the causative agent of the highly contagious mousepox disease with a high mortality rate and pox skin lesions. ECTV infection is considered today as one of the most relevant small animal models for smallpox caused by variola virus (VARV) [11–13]. ECTV is a natural mouse pathogen with a low respiratory (or dermal) lethal dose (1–100 plaque forming units (pfu)) and it can be detected in respiratory gases during preexanthem period [11, 13].

The polarized type 1 response coupled with a strong cellular response is the principal immune forces responsible for recovery from a primary ECTV infection [14]. Resistant strains (such as C57BL/6, SKH1, and AKR) show more rapid, stronger, and sustained cytolytic responses of the NK cells and cytotoxic T-lymphocytes (CTLs), compared with susceptible strains, BALB/c, DBA/2, and C3H/He [15–17]. CD8+ CTL kill targets mainly by releasing perforin and granzymes, such as granzymes A and B (GzA, GzB), through granule exocytosis [18]. Fang et al. (2012) have shown that perforin-dependent CD4+ T cell killing of ECTV-infected cells is also an important mechanism of antiviral defense [19]. Previous works suggested that CTL and NK cells can also kill target cells via the Fas/FasL pathway, which signals through caspase-8 [5]. However, ECTV encode CrmA/SPI-2 (cytokine response modifier/serine protease inhibitor), which can inhibit in vitro caspase-1 and caspase-8 but not granzyme B [20].

We have previously shown that during in vivo ECTV infection, infected cells in the central nervous system (microglia and monocytes and astrocytes and oligodendrocytes) upregulate expression of Fas/FasL, while Fas is upregulated also on the surrounding, noninfected cells [21]. Also, Fas/FasL expression in conjunctiva during ECTV infection of BALB/c mice is involved in elimination of migrating Fas+ cells but also plays an important role in the regulation of the local inflammatory reaction [22].

Increasing evidence accumulates on Fas receptor as a mediator of apoptosis-independent processes such as induction of activating and proinflammatory signals [5, 9, 23]. In this study, we investigated antiviral and inflammatory response during ECTV lung infection in Fas- and FasL-deficient and C57BL6 wild-type mice. Our results showed that lack of Fas or FasL expression led to increased viral titers and inflammatory response in lungs, followed by decreased migration of IFN-*γ* expressing NK cells and CD4+ and CD8+ T cells and disturbances in CXCL1, CXCL9, IL-10, IL-15, and TGF-*β*1 expression. Interestingly, experiments in vitro demonstrated that ECTV-infected cultures of epithelial cells, but not macrophages, upregulate Fas and FasL and are susceptible to Fas-induced apoptosis. Furthermore, lack of Fas-FasL interaction in ECTV-infected cocultures can lead to disturbances in proinflammatory chemokines production. This study shows that the Fas/FasL pathway during ECTV infection of the lungs plays an important role in controlling local inflammatory response and mounting of antiviral response.

## 2. Materials and Methods

### 2.1. Virus

ECTV-MOS strain (ATCC, VR-1374) was grown and titrated in African green monkey kidney cells (GMK-AH1) and prepared by one cycle of freeze-thawing and subsequent removal of cellular debris by centrifugation [24]. The aliquots of the stock (10^6^ PFU/mL) were snap-frozen and stored in liquid nitrogen.

### 2.2. Cell Lines

Murine epithelial Hepa 1–6 cells (ATCC CRL-1830) were maintained in Dulbecco modified Eagle medium (D-MEM) with 10% heat-inactivated fetal bovine serum (HI-FCS), 1% L-glutamine, penicillin (100 U/mL), and streptomycin (100 *μ*g/mL) (Gibco by Life Sciences Technologies, Carlsbad, CA, USA). African green monkey kidneys (GMK-AH1) were a gift from the Swedish Institute for Infectious Disease Control, Stockholm [25], and were grown in Eagle's minimum essential medium with alpha modification (*α*-MEM) supplemented with 10% heat-inactivated fetal bovine serum (HI-FCS), 1% L-glutamine, penicillin (100 U/mL), and streptomycin (100 *μ*g/mL) (Life Sciences Technologies). Hepa 1–6 cells were infected with ECTV-MOS at MOI = 2, incubated for 24 hours, and then harvested by trypsinisation.

### 2.3. Mice and Infection

Female mice, 6- to 8-week old, were used for all experiments. B6. MRL-Faslpr/J (Fas−) and B6Smn.C3-Faslgld/J (FasL−) mice were purchased from the Jackson Laboratory (Bar Harbor, ME, USA) and a breeding colony was maintained at the Oncology Centre (Warsaw, Poland) animal facilities. C57BL/6 mice were purchased from the Mossakowski Medical Research Centre (Warsaw, Poland) and used as wild-type controls. This study was performed in accordance with recommendations of the Polish Act of 21 January 2005 on animal experiments (OJ number 33, item 289) and Directive 2010/63/EU of the European Parliament and the Council of 22 September 2010 on the protection of animals used for scientific purposes. The protocol was approved by the 3rd Local Committee on the Ethics of Animal Experiments in Warsaw, Poland (Permit number: 05/2012). One day prior to challenge with ECTV, individual mice were weighed and assigned to treatment groups of 10 to 20 mice. On the day of challenge, mice were anesthetized with 0.1 mL/10 g body weight of ketamine HCl (9 mg/mL) and xylazine (1 mg/mL) by intraperitoneal injections. Anesthetized mice were laid on their dorsal side and ECTV was diluted in PBS to the required dose (5 × 10^3^ PFU) slowly loaded intranasally (10 *μ*L/mouse). Mice were observed every day for morbidity and mortality.

### 2.4. Lung Cell Isolation

At 3rd, 7th, 10th, and 14th days after infection (d.p.i.), control mice were anesthetized and lungs were isolated after perfusion with PBS without magnesium or calcium to minimize contamination with blood cells. The lungs were finely minced and then suspended in RPMI 1640 (Life Sciences Technologies) containing collagenase-dispase (1 *μ*g/mL) (Roche, Indianapolis, IN, USA). The tissues were incubated at 37°C while being mixed on an orbital shaker for 90 min. After incubation, the digestion mixtures were passed through a 100 *μ*m nylon mesh to remove undigested tissue and washed in PBS/1% FBS.

### 2.5. Apoptosis and Apoptotic Proteins Detection

Apoptosis in the single cell suspensions was detected using Annexin V-Apoptosis Detection Kit I (BD Biosciences, Franklin Lakes, NJ, USA), according to the manufacturer's protocol. The annexin V-positive, propidium iodide negative cells were scored as apoptotic cells, while all propidium iodide positive cells were considered to be necrotic. For detection of Fas and FasL, cells were washed in 1% FBS/PBS and then FITC-conjugated hamster anti-mouse Fas antibody (Jo-2) and PE-conjugated hamster anti-mouse FasL antibody (MFL4) were used (BD Biosciences). For all stainings, appropriate isotype control antibodies were used. All stainings were analyzed at the FACS Calibur (BD Biosciences) with CellQuest software (Le Pont De Claix, France).

### 2.6. Cell Characterization by Flow Cytometry

Cell suspensions were pretreated with the Fc receptors block rat anti-CD16/32 antibody (2.4G2) (BD Biosciences) according to the manufacturer's protocol. T cells were detected using rat anti-CD3e-FITC or PerCP (145-2C11), rat anti-CD4-PE (RM4-5), or rat anti-CD8-PE (53-6.7.) antibodies; NK cells were detected with rat anti-NK1.1-APC (PK136) antibody (BD Biosciences). Alveolar macrophages and inflammatory monocytes were detected using hamster anti-CD11c-APC (HL3), rat anti-CD11FITC antibody (M1/70), and rat anti-I-A-PE (M5/114.15.2) antibodies (BD Biosciences). The intracellular stainings for cytokines were carried out using the Cytofix/Cytoperm Fixation/Permeabilization Kit with BD GolgiPlug (Brefeldin A) (BD Biosciences) and monoclonal rat anti-IFN-*γ*-APC (XMG1.2), monoclonal rat anti-IL-10-FITC (JES5-16E3) (BD Biosciences), polyclonal rabbit anti-MIG (N-16; CXCL9), polyclonal goat anti-GRO (CXCL1/2), and polyclonal goat anti-IL-15 (L-20) (Santa Cruz) antibodies. Following incubation with primary antibodies, appropriate anti-rabbit PE or FITC-conjugated anti-goat and anti-rabbit antibodies were used, where necessary (BD Biosciences). For all phenotyping, rat IgG2a, rat IgG2b, and hamster IgG1 isotype antibodies conjugated with appropriate fluorochromes were used (BD Biosciences). The stained cell suspensions were analyzed in FACS Calibur for positively stained cells. The total cell count was set at 100.000 events per each experiment. All experiments were repeated 5 times. The total cell counts per experiment mean the mean of five separate experiments, each performed in a group of 5 mice with stainings performed in triplicate.

### 2.7. Histopathology of the Lung Tissue

Lungs were removed either at the indicated time points (3rd, 7th, 10th, and 14th d.p.i. and uninfected controls) or when animals had to be sacrificed during an experiment. The tissues were fixed in 4% paraformaldehyde in PBS for 24 h and then dehydrated and embedded in paraffin and cut into 6 *μ*m sections on a microtome. The sections were further stained with Harris's hematoxylin solution (Sigma Aldrich, St. Louis, MO, USA). The stained sections were dehydrated in graded series of ethanol followed by xylene and mounted with DPX (Sigma Aldrich). The images were captured with camera-equipped Zeiss AxioImager.M2 microscope using ZEN 2011 software.

### 2.8. Bone Marrow Derived Macrophages and Cocultures with Epithelial Cells

Primary cultures of bone marrow derived macrophages (BMMFs) were prepared by culturing bone marrow cells isolated from B6. MRL-Faslpr/J (Fas−) and B6Smn.C3-Faslgld/J (FasL−) and C57BL6 mice strains in the D-MEM (Life Sciences Technologies) culture medium supplemented with 25 ng/mL M-CSF (Sigma-Aldrich), 4500 mg/L glucose, antibiotics (penicillin and streptomycin), L-glutamine, and 10% fetal bovine serum (Life Sciences Technologies) for 5 days. At day 5 primary cultures of macrophages were washed and subjected to infection with ECTV-MOS at MOI = 2, incubated for up to 24 hours, and then harvested by scraping. Cocultures with epithelial cells were prepared as follows: macrophage cultures were added to a 24 h culture of epithelial Hepa 1–6 cells and then infected with ECTV at MOI = 2, incubated for next 24 hours, and harvested by trypsinisation and scraping.

### 2.9. Quantitative Reverse Transcriptase-Polymerase Chain Reaction (RT2-PCR)

Total DNA and RNA were isolated from the lung tissues preserved in RNAlater (Sigma Aldrich) using Universal DNA/RNA/Protein Purification Kit (Eurx, Gdansk, Poland). ECTV DNA titers were quantified using SYBR Green Real-Time PCR Master Mix (Life Sciences Technologies) and the ectromelia virus specific primers: 5′-CATACAGTCACAGACACTGTTG-3′ and 5′-GAT­GCT­TTC­TAC­AGT­TGT­TGG­TA-3′ [26]. For cytokine and chemokine quantification, cDNA was reverse-transcribed from 1 *μ*g of total RNA using High Capacity RNA-to-cDNA Kit (Life Sciences Technologies) according to the manufacturer's protocol. Transcripts of CXCL1, CXCL9, IL-10, IL-15, TGF-*β*1, and GADPH were quantified using TaqMan(R) Gene Expression Assays. All PCR reactions were carried out with TaqMan Gene Expression Master Mix (Applied Biosystems, Foster City, CA, USA) using 7500 Real-Time PCR System (Applied Biosystems) according to the manufacturer's protocol. Average threshold cycle (CT) values from the triplicate PCR reactions were normalized against the average CT values for GADPH from the same cDNA sample.

### 2.10. Measurement of Cytokines

Concentrations of cytokines and chemokines from culture supernatants were determined using Mouse Th1/Th2 Cytometric Bead Array (CBA) and MIG Flex Set reagents (BD Biosciences) and Mouse CXCL1 ELISA Kit (Sigma-Aldrich) according to the manufacturer's protocol. The results are presented as the means of assays performed in triplicate. The CBA data were analyzed using FCAP Array 1.0.1 and BD Cytometric Bead Array 1.4 software assay.

### 2.11. Statistical Methods

Quantitative data were presented as means ± SEM. For normal distribution of values, statistical comparisons were performed using Student's *t*-test. For data following non-Gaussian distributions, nonparametric Wilcoxon test was applied. Kaplan-Meier method and the log-rank test were used to analyze the difference between survival curves. In every analysis values of *P* ≤ 0.05 were considered significant.

## 3. Results

### 3.1. Lack of Fas or FasL during ECTV-MOS Infection Results in an Increased Infection Burden and Inflammatory Reaction

**Figure 1:**
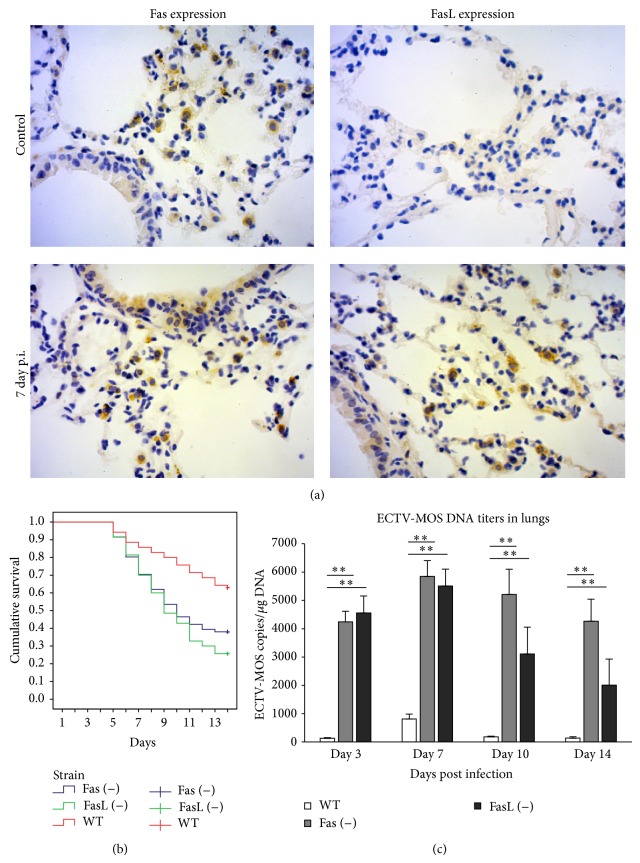
Lack of Fas and FasL expression results in an increased infection burden during mousepox infection. (a) Fas and FasL expression in the lungs of C57BL/6 mice uninfected and ECTV-infected at the 7th d.p.i. Brown color indicates a positive reaction (Fas+ or FasL−), while blue color corresponds with hematoxylin positive nuclei. (b, c) C57BL/6 (WT), B6. MRL-Faslpr/J (Fas−), and B6Smn.C3-Faslgld/J (FasL−) mice (*n* = 70 in each group) were infected with 1 × 10^3^ PFU of ECTV-MOS and monitored for 14 days. Kaplan-Meier survival analysis using the log-rank test (b) and (c) ECTV titers in lungs. Asterisks along the lines indicate statistical differences between the mice strains. The bars represent the mean from 5 separate experiments ± SEM.  ^**^Significant differences with *P* ≤ 0.001 in comparison to Fas- and FasL-deficient mice.

**Figure 2:**
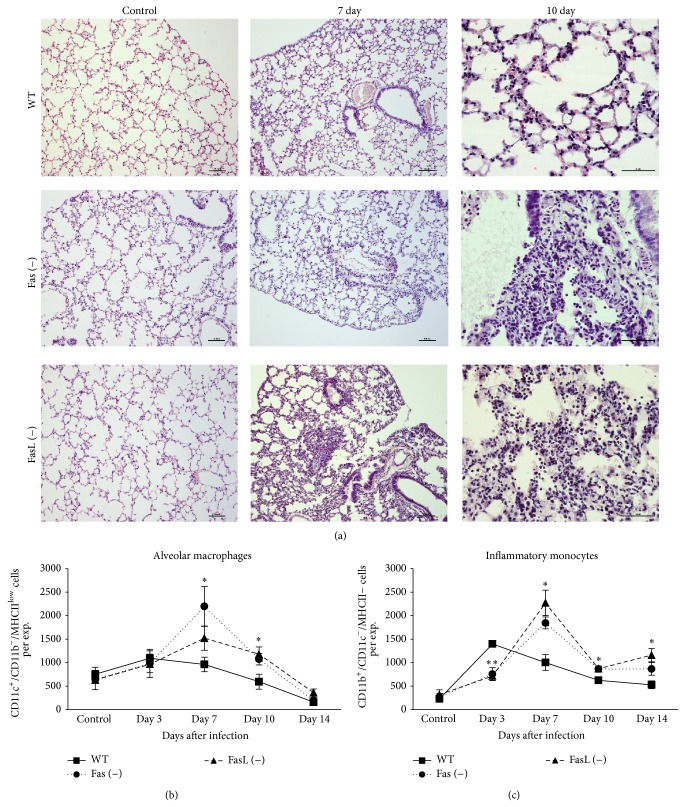
Lack of Fas or FasL leads to disturbances in the inflammatory response. (a) Histological analysis of lungs isolated from ECTV-infected Fas (−), FasL (−), and WT (C57BL/6) mice at 7 and 10 days of infection and uninfected controls. The nuclei were counterstained with Harris haematoxylin (violet) (×40 and 100). Total counts of alveolar macrophages (b) and inflammatory monocytes (b) in cell suspensions prepared from the lungs isolated from Fas (−), FasL (−), and WT (C57BL/6) mice at 3, 7, 10, and 14 days of ECTV infection and from control, uninfected mice. The bars represent the mean from 5 separate experiments ± SEM.  ^*^Significant differences with *P* ≤ 0.05 and ^**^ *P* ≤ 0.01 in comparison to wild-type mice.

To study the involvement of Fas-dependent pathway during ECTV infection of lungs, we used a well-established model of intranasal infection of C57BL6 mice. In the lungs of uninfected C57BL6 mice, Fas expression was detected on the epithelial cells of bronchial epithelium and single alveolar macrophages, while FasL expression was undetectable (Figure 1(a)). During the peak of ECTV infection in the lungs at day 7 after infection (p.i.), Fas expression was found on the bronchial epithelial cells but predominantly on the alveolar macrophages in the area surrounding bronchia, while FasL-positive cells were detected as mostly of monocyte and epithelial origin (Figure 1(a)). To elucidate the role of Fas/FasL pathway during ECTV infection, we infected Fas- and FasL-deficient mice intranasally with 5 × 10^3^ PFU of ECTV-MOS, which was about a 60% lethal dose for C57BL6 mice (WT). Mice of all three tested strains showed mortality already at day 5 p.i., however, later during infection significantly more Fas (−) and FasL (−) mice died in comparison to the wild-type strain (*P* ≤ 0.001) (Figure 1(b)). RT^2^-PCR method was used to measure ECTV DNA titers in the lungs collected at 3rd, 7th, 10th, and 14th d.p.i.; the results showed significantly increased titers of ECTV in the lungs of Fas- and FasL-deficient mice at all tested time points (*P* ≤ 0.05) (Figure 1(c)). The highest viral titers in the lungs of wild-type mice were detected at 7th d.p.i. but the titers of ECTV in Fas (−) and FasL (−) mice were significantly higher in comparison to wild-type mice during all tested period (*P* ≤ 0.001) (Figure 1(c)). Histopathologic examination of the lung tissue isolated from all tested mice strains at day 7 of ECTV infection revealed inflammatory and necrotic lesions in the epithelia of lung bronchioles (Figure 2(a)). However, the lungs of Fas- and FasL-deficient mice showed more inflammatory lesions in the area surrounding bronchia (Figure 2(a)). The inflammatory lesions observed in Fas- and FasL-deficient mice were necrotic and still present at day 10 of infection in comparison to the lung tissue of wild-type mice (Figure 2(a)). To investigate the kinetics and extent of the inflammatory reaction in lungs, we prepared single cell suspensions and analysed by flow cytometry for the total counts of alveolar macrophages and inflammatory monocytes (Figures 2(b) and 2(c)). The total numbers of alveolar macrophages (CD11c^+^/CD11b^−^/MHCII^low^) in the lungs of all tested strains increased significantly at 3rd and 7th d.p.i., to subsequently decrease at 14th d.p.i. in comparison to uninfected control mice (*P* ≤ 0.05) (Figure 2(b)). When comparing to ECTV-infected wild-type mice, both Fas- and FasL-deficient mice at 7th and 10th d.p.i. showed significantly increased total counts of alveolar macrophages (*P* ≤ 0.05) (Figure 2(b)). Assessment of inflammatory monocytes (CD11b^+^/CD11c^−^/MHCII−) in the lungs of ECTV-infected mice revealed that the total counts of inflammatory monocytes were significantly increased during the whole infection period (*P* ≤ 0.05) (Figure 2(c)). However, the wild-type mice showed significantly higher total counts of inflammatory monocytes at 3rd d.p.i. in comparison to Fas- and FasL-deficient mice (*P* ≤ 0.001) (Figure 2(c)). Later during infection (7th, 10th, and 14th d.p.i.), Fas- and FasL-deficient mice showed an opposite effect with a more significant inflammatory reaction in comparison to wild-type mice (*P* ≤ 0.05) (Figure 2(c)).

### 3.2. Lack of Fas or FasL during ECTV Infection Impairs the Antiviral Response in Lungs

**Figure 3:**
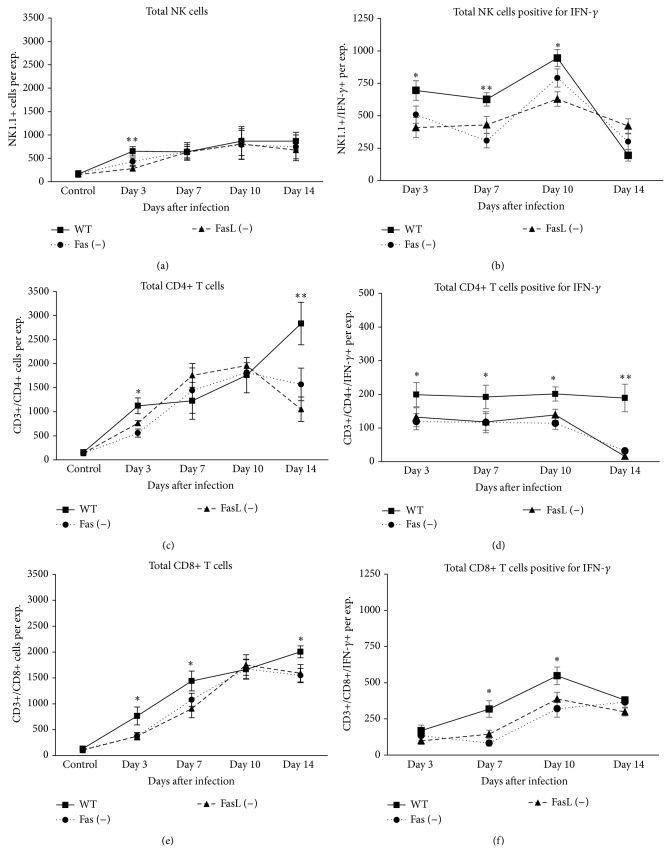
Lack of Fas or FasL leads to disturbances in antiviral immune response. Total counts of NK cells (a) NK/IFN-*γ*+ cells (b), CD4+ T cells (c), CD4+/IFN-*γ*+ T cells (d), CD8+ T cells (e), and CD8+/IFN-*γ*+ T cells (f) in cell suspensions prepared from the lungs isolated from Fas (−), FasL (−), and WT (C57BL/6) mice at 3, 7, 10, and 14 days of ECTV infection and from control, uninfected mice. The bars represent the mean from 5 separate experiments ± SEM.  ^*^Significant differences with *P* ≤ 0.05 and ^**^ *P* ≤ 0.01 in comparison to Fas- and FasL-deficient mice.

To determine the antiviral response, we measured total counts not only of NK1.1 (NK1.1+/CD3−) cells but also of CD4+ and CD8+ T cells in the lungs of C57BL/6, Fas (−), and FasL (−) mice at 3rd, 7th, 10th, and 14th d.p.i. (Figure 3). We found that lungs of wild-type mice showed significantly increased total counts of NK cells, CD4+, and CD8+ T cells at 3rd d.p.i in comparison to Fas- and FasL-deficient mice (*P* ≤ 0.05) (Figures 3(a), 3(c), and 3(e)). Furthermore, ECTV-infected wild-type mice showed significantly increased total counts of CD8+ T cells at 7th d.p.i. (*P* ≤ 0.05) (Figure 3(c)) and of CD4+ and CD8+ T cells at 14th d.p.i. (*P* ≤ 0.05) (Figures 3(c) and 3(e)). Given the role of IFN-*γ* in the early antiviral response, we next tested for the total counts of NK, CD4+, and CD8+ T cells expressing IFN-*γ* in the lungs (Figures 3(b), 3(d), and 3(f)). When comparing to ECTV-infected Fas- and FasL-deficient mice, wild-type mice showed significantly increased total counts of IFN-*γ* expressing NK cells at 3rd, 7th, and 10th d.p.i. (*P* ≤ 0.05) (Figure 3(b)). Furthermore, the lungs of wild-type mice revealed significantly increased total counts of CD4+ expressing IFN-*γ* during the whole infection period in comparison to Fas- and FasL-deficient mice (*P* ≤ 0.05) (Figure 3(d)). For the CD8+ T cells expressing IFN-*γ*, wild-type mice showed significantly higher total counts of these cells at 7th and 10th d.p.i. in comparison to Fas- and FasL-deficient mice (*P* ≤ 0.05) (Figure 3(f)).

**Figure 4:**
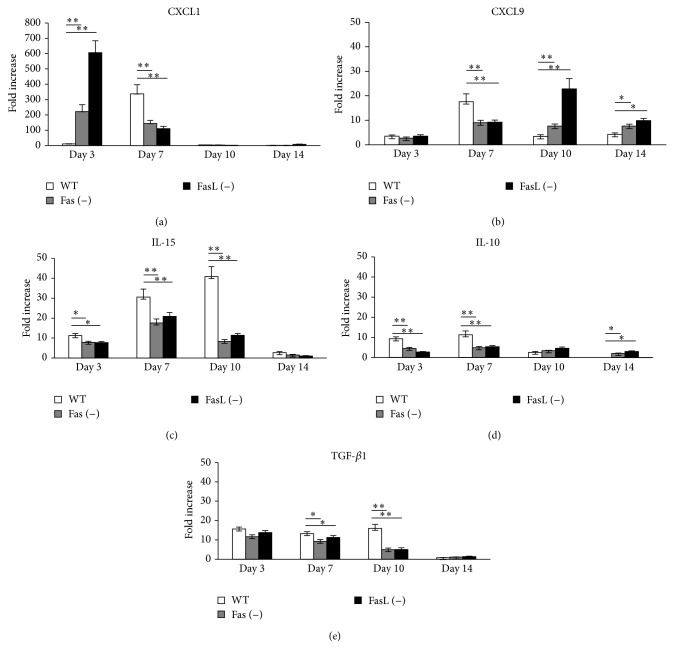
Lack of Fas or FasL during mousepox infection results in disturbances of cytokine and chemokine production. Relative mRNA levels of CXCL1, CXCL9, IL-10, IL-15, and TGF-*β*1 in the lungs isolated from Fas (−), FasL (−), and WT (C57BL/6) mice at 3, 7, 10, and 14 days of ECTV infection and from control, uninfected mice. The mRNA expressions were normalized by that of GAPDH, and the lungs of control, uninfected mice were estimated as 1. The bars represent the mean from 3 separate experiments ± SEM.  ^*^Significant differences with *P* ≤ 0.05 and ^**^ *P* ≤ 0.01.

Taking into account the role of cytokines and chemokines in mounting an early antiviral response we tested for the levels of CXCL1, CXCL9, IL-10, and IL-15 mRNA expression in the lungs of all three mice strains during the whole ECTV infection period (Figure 4). Quantification of relative mRNA of CXCL1 in Fas- and FasL-deficient mice showed significantly increased mRNA levels of this cytokine at 3rd d.p.i. (*P* ≤ 0.001) and significantly decreased CXCL1 mRNA levels at 7th d.p.i. in comparison to wild-type mice (*P* ≤ 0.001) (Figure 4(a)). On the contrary, the levels of CXCL9 at 3rd d.p.i. ECTV infection were significantly increased in the lungs obtained from wild-type mice in comparison to Fas- and FasL-deficient mice (*P* ≤ 0.001) (Figure 4(b)), while at 7th d.p.i. we observed an opposite effect in the lungs of ECTV-infected Fas- and FasL-deficient mice (*P* ≤ 0.001) (Figure 4(b)). Many authors suggested an important role of IL-15 in mobilization of NK cells to the orthopoxvirus infected organs [27]. Following the data obtained for NK cells (Figure 3), also we observed significantly decreased expression levels of IL-15 in Fas- and FasL-deficient mice at 3rd, 7th, and 10th days of ECTV infection (Figure 4(c)). To check if the lack of Fas or FasL expression may influence production of anti-inflammatory cytokines, we tested for IL-10 and TGF-*β*1 mRNA levels in the lungs during ECTV infection. The Fas- and FasL-deficient mice showed significantly decreased levels of IL-10 expression early during infection (3rd and 7th d.p.i) in comparison to wild-type mice (*P* ≤ 0.001) (Figure 4(d)), followed by significantly decreased mRNA levels of TGF-*β*1 at 7th and 10th d.p.i. (*P* ≤ 0.05) (Figure 4(e)). In contrast, significantly increased IL-10 mRNA levels were observed in Fas- and FasL-deficient mice at 14th d.p.i (*P* ≤ 0.05) (Figure 4(d)). The results obtained by RT-PCR analysis were confirmed by staining of the percentage of cells positive for IL-10, IL-15, CXCL1, and CXCL9 in the cell suspensions prepared from ECTV-infected and uninfected control lungs (Table 2).

### 3.3. ECTV-Infected Monocytes and Epithelial Cells Show Different Response to Fas-Induced Apoptosis

**Figure 5:**
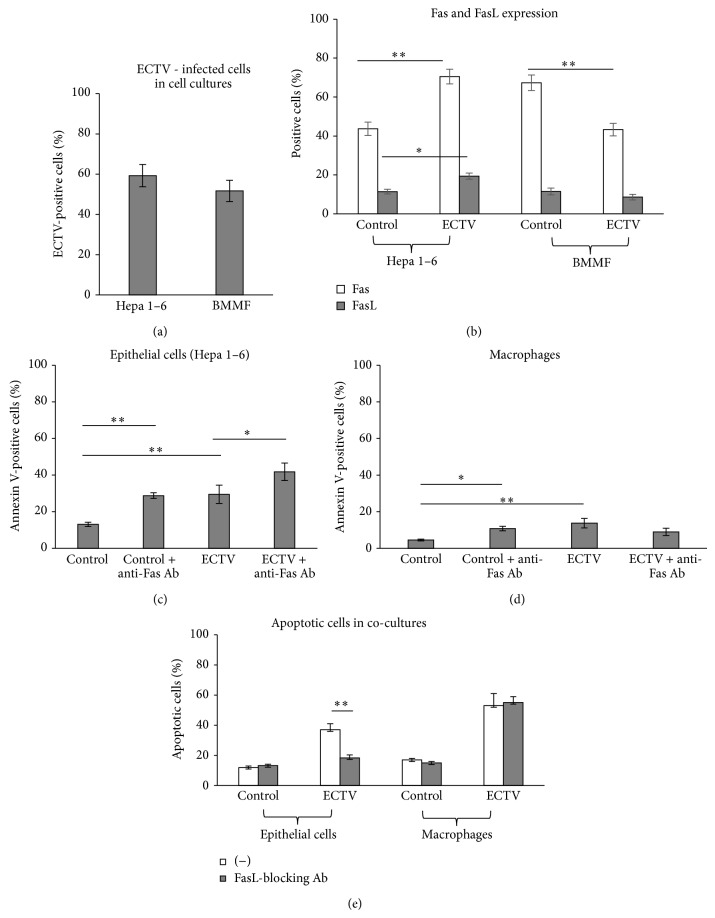
ECTV-infected macrophages and epithelial cells show different response to Fas-induced apoptosis. (a) Percentages of ECTV-infected cells in Hepa 1–6 epithelial cell cultures and bone marrow derived macrophages (BMMF) at 24 h p.i. (b) Percentage of Fas- and FasL-positive cells in epithelial Hepa 1–6 and BMMF cultures at 24 h of ECTV infection. Percentage of apoptotic (annexin V-positive) cells in epithelial Hepa 1–6 (c) and BMMF cultures (d), infected or not with ECTV and exposed or not to anti-Fas cytotoxic antibody (10 mg/mL). (e) Percentage of apoptotic cells in the cocultures of epithelial Hepa 1–6 and macrophage cells infected or not with ECTV and exposed to anti-FasL-blocking antibody (10 *μ*g/mL). The bars represent the mean from 3 separate experiments (*N* = 3) ± SEM.  ^*^Significant differences with *P* ≤ 0.05 and ^**^ *P* ≤ 0.01.

To study the role of Fas-dependent apoptotic response during ECTV infection, we employed an in vitro model consisting of mouse Hepa 1–4 epithelial cell culture and bone marrow derived macrophage cultures (BMMF) infected with ECTV for 24 h. Both cell cultures infected with MOI = 2 of ECTV showed approximately 60% of infected cells (Figure 5(a)). Upon infection with ECTV, epithelial cell cultures significantly upregulated Fas and FasL expression (*P* ≤ 0.05) (Figure 5(b)), while mouse macrophages infected with ECTV significantly decreased Fas expression (*P* ≤ 0.001) (Figure 5(b)). To assess the sensitivity of ECTV-infected epithelial cells and macrophages to Fas-induced apoptosis we used an anti-mouse Fas cytotoxic antibody (Jo-1 clone). In both the Hepa 1–6 epithelial cell cultures and macrophages, addition of the anti-Fas antibody to uninfected cultures resulted in an increased percentage of annexin V-positive cells after 24 h (*P* ≤ 0.05) (Figures 5(c) and 5(d)). Addition of anti-Fas antibody to ECTV-infected cultures significantly increased Fas-mediated apoptosis but only in epithelial cells (*P* ≤ 0.05) (Figures 5(c) and 5(d)). In order to test the role of Fas-induced apoptosis in the lungs during ECTV infection we constructed a model of epithelial cells cocultured with macrophages in the presence of FasL-blocking antibody (clone MFL4) (Figure 5(e)). At 24 h of infection in the presence of FasL-blocking antibody, only epithelial cells showed a significant decrease in the percentage of apoptotic cells (*P* ≤ 0.001) (Figure 5(e)). ECTV infection of cocultures led to a significant decrease in production of TNF-*α*, CXCL1, and CXCL9 (*P* ≤ 0.05) (Table 1). However, blocking of Fas-FasL interaction between macrophages and epithelial cells further downregulated CXCL1 and CXCL9 production (*P* ≤ 0.05) (Table 1).

## 4. Discussion

Fas-FasL signalling is involved in apoptosis of immune cells as well as of the virus infected target cells. However, evidence accumulates on Fas as a mediator of apoptosis-independent processes including proliferation, angiogenesis, fibrosis, and inflammation. The goal of this study was to investigate the role of the Fas pathway in regulation of inflammation and mounting antiviral response during mousepox lung infection of mice. In the present study, we found that lack of Fas-FasL signalling leads to disturbances in the induction of the local antiviral cytokine environment during lung mousepox infection and delayed clearance of the local inflammatory reaction.

As a natural mouse pathogen, ECTV offers the unique opportunity to study natural course of virus-host interaction. This virus is very similar to variola virus (VARV), the agent of human smallpox, the zoonotic monkeypox virus, and the smallpox vaccine species vaccinia virus (VACV) [13, 28]. Following an intranasal infection with a relatively small dose of virus, ECTV infects the upper and lower respiratory tract although early during infection there is no obvious lung involvement. Furthermore, virus can be detected in respiratory gases during the secondary viraemia in the preexanthem period [13, 28]. Although ECTV infects all mouse strains, the outcome of primary infection varies depending on the strain, dose, and route of infection with BALB/c strain being the most susceptible. In resistant strains, such as C57BL/6 (B6), the replication of the virus is controlled by the combined action of innate and adaptive immune mechanisms [28].

Cytotoxic T-lymphocytes (CTLs) and natural killer (NK) cells are key components of the host immune system protecting against viruses. CTLs cells employ two major mechanisms to induce apoptosis of target infected cells: one is via the Fas/FasL pathway and the other is via the granule exocytosis pathway [18]. Furthermore, during a late phase after the infection, FasL/Fas signaling is essential for elimination of activated peripheral lymphocytes to terminate inflammation [29]. Recently, it has been demonstrated that FasL-deficient B6Smn.C3-Tnfsf6gld/J mice are more resistant to lethal influenza virus infection than C57Bl/6J mice [30]. Other studies also demonstrated that activation of Fas signaling may cause acute lung inflammation [31]. These findings indicate the role of FasL/Fas signaling in pathogenesis of lung inflammatory reaction.

In this study we observed that Fas- and FasL-deficient mice showed significantly higher mortality and virus titers in lungs (Figure 1), indicating the role of Fas/FasL – dependent apoptotic pathway in the mounting of antiviral response. Like other orthopoxviruses, ECTV is able to inhibit death receptor-induced apoptosis by expression of SPI-2, a serine protease inhibitor (serpin) [20, 32]. Transient expression of SPI-2 protected cells from tumour necrosis factor-mediated apoptosis and inhibited the activity of caspases 1 and 8 but not caspases 3 and 6 or granzyme B [20, 32]. Apoptotic cell death induction by gzmA and/or gzmB is sufficient for the recovery of mice from ECTV infection and cannot be inhibited by virus-encoded proteins [32]. NK cells are innate effector cells serving as a first line of defense against certain viral infections and tumors, capable of producing IFN-*γ* [33]. NK cells have been demonstrated to be important in recovery from mousepox and resistant C57BL/6 mice mount a strong NK cell response [34, 35], which further allows for the development of antiviral CD8+ T cell and B cell responses.

In this study we observed that early during ECTV infection, Fas- and FasL-deficient mice showed significantly lower numbers of NK cells in lungs (Figure 3). Also the lungs of wild-type mice were characterized with the increased total counts of NK cells expressing IFN-*γ* (Figure 3) in comparison to Fas- and FasL-deficient mice early and later during infection. Numerous studies have supported the critical importance of IFN-*α*/*β* and IFN-*γ* in recovery from ECTV infection [36]. The activity of IFN-*γ* can be inhibited at the level of receptor engagement by ECTV vIFN-cR, which shows only 20% sequence similarity to the host receptor [37]. Many authors have shown that CD4+ T cell help-independent CD8+ T cell responses are essential for the early control of primary ECTV infection, while CD4+ T cell help-dependent Ab responses are essential for the late infection clearance [38]. However, Fang et al. (2012) reported that the CD4+ T cell responses to ECTV are also important and the responding CD4+ T cells show in vivo perforin-dependent, MHC II-restricted CTL activity, which directly contributes to controlling the virus [19]. In our work we found that the lungs of Fas (−) and FasL (−) mice mobilized significantly less total numbers of CD4+ T cells at 3rd and 14th day of ECTV infection in comparison to wild-type mice and the total numbers of CD4+ T cells positive for IFN-*γ* were significantly lower during the whole infection period (Figure 3). Similarly, we observed significantly decreased total counts of CD8+ T cells early and later during infection, followed by significantly decreased numbers of CD8+/IFN-*γ* in ECTV-infected Fas- and FasL-deficient mice (Figure 3).

We have previously demonstrated that lack of Fas or FasL expression disturbs mounting of the antiviral response during vaginal HSV-2 infection due to decreased production of CXCL9 and IFN-*γ* and increased production of IL-10 and TGF-*β*1 [39, 40]. Here, we also observed disturbances in the pattern of cytokine and chemokine production in the lungs of Fas- and FasL-deficient mice during ECTV infection (Figure 4). CXC type chemokines, including CXCL9 and CXCL10, are potent chemoattractants for activated T cells, NK cells, monocytes, dendritic cells, and B cells [41]. Also IL-15 has been suggested to be important in mobilization of NK cells to the orthopoxvirus infected organs [27]. Here, we observed that early during ECTV infection wild-type mice showed much more potent expression of CXCL9 at 7th d.p.i. and IL-15 in lungs during almost all tested period in comparison to Fas- and FasL-deficient mice. Later during infection, the lungs of Fas- and FasL-deficient mice expressed significantly more CXCL9, which can reflect increased migration of alveolar macrophages and inflammatory monocytes (Figure 2).

It has been suggested that, under conditions of chronic infection, IL-10 may limit tissue damage associated with a persistent inflammatory state [42], while TGF-*β*1 is important in regulation of the local immune response [43]. Here, we found that the excessive inflammatory reaction observed in the lungs of Fas- and FasL-deficient mice later during ECTV infection was accompanied by significantly decreased expression of IL-10 and TGF-*β*1 (Figure 4).

Starting from day 7 of ECTV infection, lungs of Fas- and FasL-deficient mice were characterized with the significant inflammatory reaction expressed as increased total counts of alveolar macrophages and inflammatory monocytes (Figure 2). This reaction resulted in the presence of inflammatory lesions of necrotic character. CXCL1 specifically targets neutrophils through the receptor CXCR2 promoting chemotaxis and activation of neutrophils [44] but despite the initial significant increase of CXCL1 expression in Fas- and FasL-deficient mice at 3rd day of ECTV infection, later during infection we observed an opposite effect (Figure 4).

During lung inflammation, inflammatory monocytes migrate from the circulation to the airspaces. As inflammation resolves, macrophage numbers decrease and normal tissue structure and function are restored. Kitamura et al. (2001) suggested that Fas plays a major role in determining infiltrating macrophage cell fate. Recruited macrophages express high levels of Fas on their surface and the administration of a Fas activating antibody led to their depletion [45]. High levels of both the soluble and the membrane-bound forms of FasL are present in the lungs during inflammation [45] and the potential sources of FasL in the lungs include inflammatory macrophages, activated lymphocytes, and the epithelial cells [45]. In this study, ECTV-infected macrophages decreased Fas expression, while ECTV-infected epithelial cells increased both Fas and FasL expression in vitro. Furthermore, ECTV-infected epithelial cells but not macrophages were susceptible to Fas-induced apoptosis (Figure 5). During ECTV infection in vivo, the source of FasL consisted of infiltrating lymphocytes, macrophages, and the epithelial cells, while upregulated Fas expression was detected on monocytes and epithelial cells (Figure 1). We can therefore hypothesize that, during ECTV infection of lungs, infiltrating activated lymphocytes and monocytes may limit the ECTV infection in Fas+ epithelial cells, while FasL− expressing epithelial cells and infiltrating lymphocytes eliminate Fas+ inflammatory monocytes. This results in elimination of the local inflammation. However, it also raises the possibility of ECTV+ macrophages surviving Fas-induced apoptosis.

We have previously shown that ECTV infection of the brain not only leads to upregulation of FasL on the surface of the infected cells but also results in upregulation of Fas on the surrounding cells, leading to apoptosis and clearance of ECTV-MOS infection and local inflammation [21]. Similarly, Fas/FasL-dependent cell turnover plays an important role in clearance of ECTV-infected epithelium, local inflammation, and further virus spread [22].

As mentioned above, Fas signaling plays an important role in production of CXCL9, CXCL10, and TNF-*α* by monocytes and CXCL1 by epithelial cells, from HSV-2 infected Fas- and FasL-deficient mice [9, 39]. In this study, mice lacking Fas or FasL also presented disturbances in CXCL1, CXCL9, IL-15, and IFN-*γ* expression during ECTV infection. Additionally, we found that Fas-induced apoptosis in ECTV-infected cocultures of macrophages and epithelial cells is also involved in production of CXCL1 and CXCL9 chemokines important in further antiviral and inflammatory response (Table 1).

In conclusion, the present study for the first time demonstrates that Fas/FasL pathway during ECTV infection of the lungs plays an important role in controlling local inflammatory response and mounting of antiviral response. These effects are associated with the role of Fas/FasL signaling not only in production of cytokines and chemokines (IFN-*γ*, CXCL1, CXCL9, IL-15, IL-10, and TGF-*β*1) but also in elimination of infiltrating inflammatory leucocytes.

## Figures and Tables

**Table 1:** Production of TNF-alpha, CXCL1, and CXCL9 in pg/mL in the supernatant of epithelial Hepa 1–6 cells and bone marrow derived macrophages (BMMF) cocultures infected or not with ECTV for 24 h and incubated or not in the presence of 10 *µ*g/mL of anti-FasL-blocking antibody (clone MFL-4). All results represent means from 3 separate experiments ± SEM.

	(−)	FasL-blocking Ab
TNF-alpha		
Control	49.5 ± 5.3	46.8 ± 5.2
ECTV	25.5 ± 3.1^*^	33.3 ± 4.02^*^

CXCL1		
Control	40.8 ± 5.1	36.7 ± 2.9
ECTV	24.5 ± 2^*^	16.8 ± 0.9^∗†^

CXCL9		
Control	45.5 ± 3.1	42 ± 4.5
ECTV	29.1 ± 1.7^*^	14.5 ± 1.3^∗†^

^*^Significant differences with *P* ≤ 0.05, by *t*-test in comparison to control, uninfected cultures. ^†^Significant difference (*P* ≤ 0.05) by *t*-test in comparison to cultures nontreated with FasL-blocking antibody.

**Table 2:** Percentages of cells expressing IL-10, IL-15, CXCL1, and CXCL9 in the lungs isolated from ECTV-infected Fas (−), FasL (−), and WT (C57BL/6) mice at 3rd, 7th, 10th, and 14th days of infection and uninfected controls. All results represent means from 3 separate experiments ± SEM.

	Control	Day 3	Day 7	Day 10	Day 14
% IL-10 expressing cells					
WT	10.23 ± 2.11	12.28 ± 2.88	11.22 ± 1.01	7.56 ± 0.34	0.0
Fas (−)	11.07 ± 2.34	5.2 ± 0.42^**^	4.82 ± 0.22^*^	8.7 ± 0.99	11.8 ± 1.78^**^
FasL (−)	8.99 ± 2.67	6.11 ± 0.41^**^	5.33 ± 0.69^*^	9.8 ± 1.2	13.1 ± 1.2^**^

% IL-15 expressing cells					
WT	0.89 ± 0.13	5.56 ± 0.44	7.23 ± 0.47	7.1 ± 0.67	1.9 ± 0.55
Fas (−)	0.94 ± 0.15	1.89 ± 0.2^*^	2.1 ± 0.09^**^	1.23 ± 0.01^*^	0.89 ± 0.31
FasL (−)	0.97 ± 0.11	1.99 ± 0.19^*^	1.7 ± 0.18^**^	1.41 ± 0.12^*^	0.79 ± 0.47

% CXCL1 expressing cells					
WT	5.23 ± 0.66	11.45 ± 2.34	28.23 ± 1.07	8.89 ± 0.76	6.54 ± 0.59
Fas (−)	4.59 ± 0.45	23.9 ± 3.1^*^	8.7 ± 1.3^**^	7.65 ± 0.8	5.49 ± 0.87
FasL (−)	6.01 ± 0.55	28.23 ± 2.66^**^	9.1 ± 1.08^**^	7.05 ± 0.69	6.3 ± 0.65

% CXCL9 expressing cells					
WT	2.7 ± 0.22	3.5 ± 0.3	16.8 ± 1.22	5.3 ± 0.43	3.7 ± 0.33
Fas (−)	2.45 ± 0.2	2.7 ± 0.44	2.51 ± 0.3 ^**^	17.69 ± 2.1^*^	8.45 ± 0.9^*^
FasL (−)	1.99 ± 0.34	3.5 ± 0.32	3.09 ± 0.23^**^	19.99 ± 2^*^	7.96 ± 0.88^*^

^*^Significant differences with *P* ≤ 0.05 and ^**^ *P* ≤ 0.01 by *t*-test in comparison to control, uninfected cultures.
